# Pharmacological inhibition of RUNX1 reduces infarct size after acute myocardial infarction in rats and underlying mechanism revealed by proteomics implicates repressed cathepsin levels

**DOI:** 10.1007/s10142-024-01391-2

**Published:** 2024-06-12

**Authors:** Hengshu Chen, Si Wang, Xiaoling Zhang, Xing Hua, Meng Liu, Yanan Wang, Simiao Wu, Weihong He

**Affiliations:** 1 Department of Neurology, West China Hospital, Sichuan University, Chengdu, 610041 China; 2 Department of Cardiology, West China Hospital, Sichuan University, Chengdu, 610041 China; 3 Department of Physiology, West China School of Basic Medical Sciences and Forensic Medicine, Sichuan University, Chengdu, 610041 China

**Keywords:** Runx1, Cathepsin, Myocardial infarction, Cardiac protection, Therapeutic target

## Abstract

Myocardial infarction (MI) results in prolonged ischemia and the subsequent cell death leads to heart failure which is linked to increased deaths or hospitalizations. New therapeutic targets are urgently needed to prevent cell death and reduce infarct size among patients with MI. Runt-related transcription factor-1 (RUNX1) is a master-regulator transcription factor intensively studied in the hematopoietic field. Recent evidence showed that RUNX1 has a critical role in cardiomyocytes post-MI. The increased RUNX1 expression in the border zone of the infarct heart contributes to decreased cardiac contractile function and can be therapeutically targeted to protect against adverse cardiac remodelling. This study sought to investigate whether pharmacological inhibition of RUNX1 function has an impact on infarct size following MI. In this work we demonstrate that inhibiting RUNX1 with a small molecule inhibitor (Ro5-3335) reduces infarct size in an in vivo rat model of acute MI. Proteomics study using data-independent acquisition method identified increased cathepsin levels in the border zone myocardium following MI, whereas heart samples treated by RUNX1 inhibitor present decreased cathepsin levels. Cathepsins are lysosomal proteases which have been shown to orchestrate multiple cell death pathways. Our data illustrate that inhibition of RUNX1 leads to reduced infarct size which is associated with the suppression of cathepsin expression. This study demonstrates that pharmacologically antagonizing RUNX1 reduces infarct size in a rat model of acute MI and unveils a link between RUNX1 and cathepsin-mediated cell death, suggesting that RUNX1 is a novel therapeutic target that could be exploited clinically to limit infarct size after an acute MI.

**Supplementary Information:**

The online version contains supplementary material available at 10.1007/s10142-024-01391-2.

## Background

Myocardial infarction (MI) is one of the leading causes of death and disability worldwide (Tsao et al. 2022). Acute blockage of coronary artery leads to prolonged ischemia and subsequent cell death. The irreversible loss of cardiomyocytes after MI initiates a reparative process involving the generation of fibrillar collagens at the infarct region (Kehat and Molkentin 2010). These structural changes are fundamental to adverse left ventricular (LV) remodeling, which manifests clinically as LV dilation, LV wall thinning, and reduced contractile function (Konstam et al. 2011). The progression of adverse LV remodeling, together with neurohumoral activation, leads to systolic heart failure which is associated with high rates of morbidity and mortality (Savarese et al. 2023). Since the size of the myocardial infarct is dependent on the amount of lost myocardium and has been strongly linked with the development of adverse LV remodelling (Bulluck et al. 2016), limiting infarct size serves as an important strategy to protect against adverse LV remodeling and prevent the onset of heart failure. Therefore, novel therapeutic targets which can be pharmacologically targeted to reduce infarct size are urgently needed to improve the quality of life and the survival rate of patients with MI.

Runt-related transcription factor-1 (RUNX1) is a member of the core-binding factor family of transcription factors involved in transcriptional regulation of gene expression (Krishnan 2023; Rozen et al. 2023). RUNX gene family is composed of three members (RUNX1, RUNX2 and RUNX3), which encode the DNA-binding (α) subunits of a family of transcription factors that orchestrate proliferation, differentiation, and cell survival in multiple lineages. The three members of RUNX family genes are all known developmental regulators, each with its own distinct tissue-specific and spatial-temporal pattern of expression (Mevel et al. 2019; Riddell et al. 2020). RUNX1 is best characterized for its involvement in hematopoiesis and blood cancer. Although the previous focus of RUNX1 research has been predominately in cancer and hematological field, recent evidence suggests that Runx1 plays a critical role in ischemic heart disease (Riddell et al. 2020). McCarroll & He et al. (McCarroll et al. 2018) demonstrated for the first time that the up-regulation of RUNX1 following MI drives adverse LV remodelling. In the previous study, we have shown that cardiomyocyte-specific Runx1-deficient mice were protected against adverse LV remodeling after MI, maintaining cardiac contractile function as demonstrated by 8-week echocardiography conducted at multiple time points. At the mechanistic level, cardiomyocytes isolated from these Runx1-deficient mice exhibited substantially improved calcium handling which was achieved through increased phosphorylation of phospholamban and relief of sarco/endoplasmic reticulum Ca^2+^-ATPase inhibition (McCarroll et al. 2018). Importantly, Runx1 expression in the heart is increased as early as 1-day following MI, at which time cardiac contractility is also markedly decreased (McCarroll et al. 2018), indicating that we can intervene by targeting RUNX1. The translational potential of RUNX1 is further confirmed by a more recent study from Loughrey’s group (Martin et al. 2023). By utilizing various approaches to inhibit RUNX1 in mice following MI, including an adenoviral vector expressing Runx1-shRNA, a cardiotropic adeno-associated virus serotype 9 (AAV9) expressing a shRNA targeting Runx1, and a small molecule inhibitor (Ro5-3335), this study showed that both gene therapeutic and pharmacological approaches to antagonize RUNX1 preserve cardiac function following MI (Martin et al. 2023). However, although this study included infarct size measurement at 7-days post-MI performed by Sirius red staining on fixed histological sections (Martin et al. 2023), the acute ischemic injury and the tissue viability following MI, which can be assessed by triphenyltetrazolium chloride (TTC) staining for fresh tissue sample (Lindsey et al. 2018), has not been examined. Since Runx1 expression is increased as early as 1-day post-MI, its impact on infarct size at the early stage of tissue injury post-MI needs to be evaluated.

The cardiomyocyte death following MI results in the generation of three different regions within LV: the infarct zone (IZ) consisting of necrotic tissue which subsequently gives rise to fibrous tissue, the remote zone (RZ) located furthest away from the IZ, and the border zone (BZ) which surrounds the IZ (Martin et al. 2023). The infarct size (size of the IZ) is an important predictor of cardiac contractile dysfunction and adverse LV remodeling (Del Buono et al. 2022; Del Buono et al. 2021). Larger infarct size is associated with greater contractile dysfunction and adverse LV remodeling (Chareonthaitawee et al. 1995). At the patient level, infarct size in MI patients undergoing primary percutaneous coronary intervention (PCI) was strongly associated with subsequent heart failure and mortality (Stone et al. 2016). The BZ, which is adjacent to the IZ, is viable but ischemically damaged. The pathophysiology of BZ is important because the surviving myocardium in the BZ is subject to intense fluctuations of its cellular microenvironment and is thought to be more susceptible to additional ischemic damage (van Duijvenboden et al. 2019). Furthermore, the viable cardiomyocytes in the BZ are hypocontractile and their dysfunction following MI seemingly expands progressively to involve contiguous myocardium, leading to adverse LV remodelling and heart failure (Jackson et al. 2002). Our previous study has shown that programmed cell death pathways can be therapeutically targeted to limit infarct size. He et al. (He et al. 2022) demonstrated activated apoptosis in cardiomyocytes obtained from ex vivo isolated rat hearts which are subjected to ischemia followed by reperfusion. Pharmacological treatment antagonizing cathepsin-L in ex vivo hearts showed effects of suppressing apoptosis and reducing infarct size (He et al. 2022).

Given that RUNX1 expression is increased in the BZ 1-day after MI and is associated with impaired cardiac function (McCarroll et al. 2018; Martin et al. 2023; Kubin et al. 2011; Gattenlohner et al. 2003), it is important to clarify whether pharmacological inhibition of RUNX1 has an impact on infarct size following acute MI. To address this gap, we used an in vivo rat model of acute MI in which RUNX1 was inhibited by a small molecule inhibitor. We report that rats treated with RUNX1 inhibitor demonstrated a reduced infarct size, with decreased cardiac cathepsin levels, indicating that antagonizing RUNX1 function may suppress cathepsin-mediated cell death. Thus, RUNX1 represents a promising therapeutic target with translational potential to limit infarct size among patients with MI.

## Results

### Inhibition of RUNX1 reduces infarct size after acute MI

**Fig. 1:**
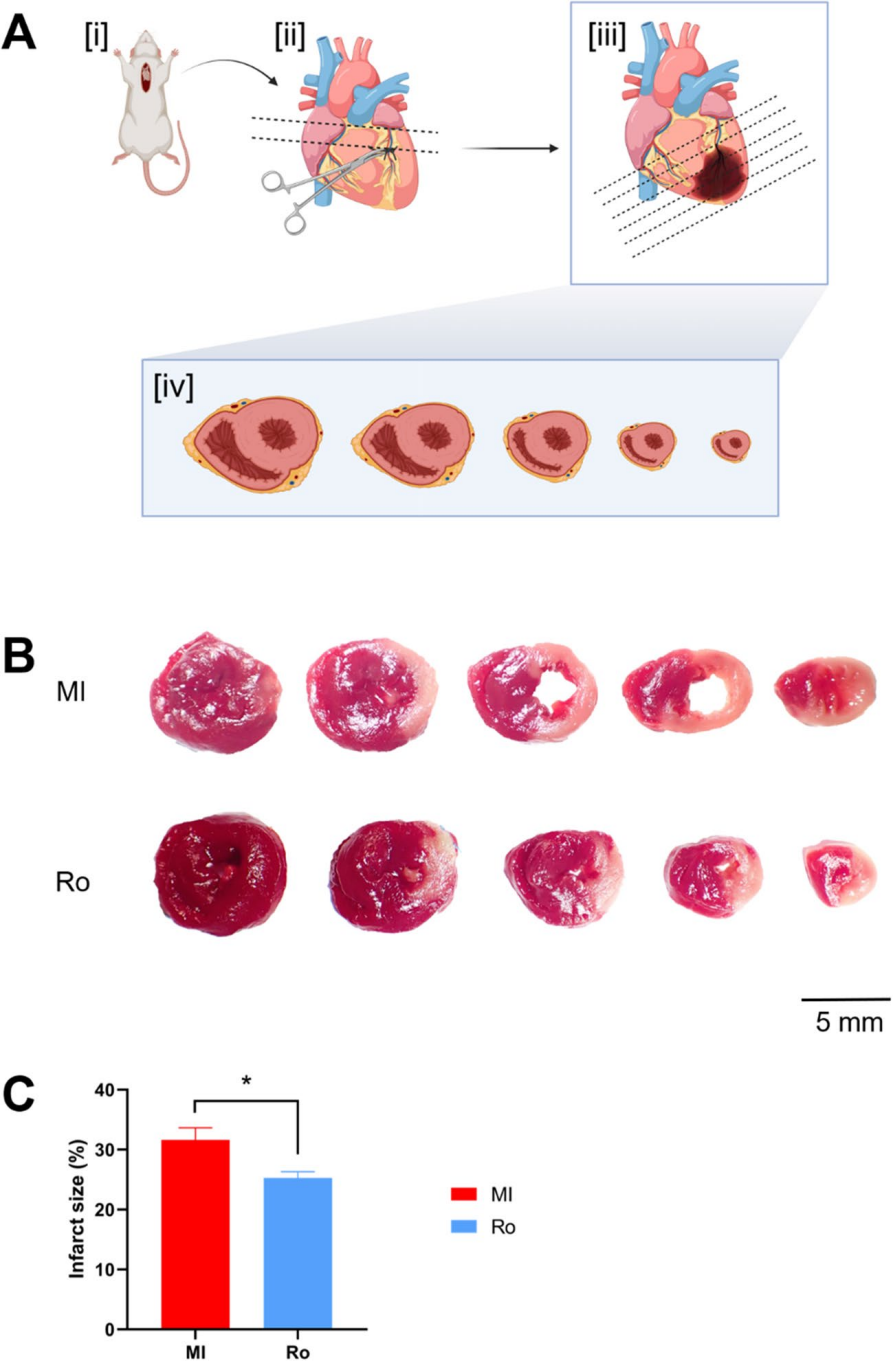
Infarct size in rat hearts treated with the RUNX1 inhibitor Ro5-3335 *in vivo*. Data were collected at 24 hours after infarction. (**A**) Schematic of the protocol used. (**B**) Representative TTC staining of heart slices. The red staining is viable tissue, and the pale color represents dead tissue (5 mm scale bar). (**C**) The mean infarct size for MI group (*n*= 8) and Ro group (*n*= 8). *P < 0.05. Study groups: MI, MI rat hearts treated by vehicle; Ro, MI rat hearts treated by Ro5-3335. Figure 1A was prepared using BioRender.com under a publication license

The primary focus of this work is determining whether pharmacologically antagonizing RUNX1 function reduces infarct size at the acute phase following MI. In the previous study, Martin et al. (Martin et al. 2023) have shown that inhibition of RUNX1 in the BZ prevents cardiac contractile dysfunction following MI. However, the beneficial effects on infarct size were inconsistent between experiments using different strategies of RUNX1 inhibition (Martin et al. 2023). Interestingly, whilst intravenous injection of a cardiotropic adeno-associated virus serotype 9 expressing a shRNA targeting Runx1 (AAV9-Runx1-shRNA) produced a reduction in infarct size at 7-days post-MI, measured by Sirius red staining on fixed heart slices, direct myocardial injection of adenovirus-mediated Runx1-shRNA did not show the same effect of reducing infarct size (Martin et al. 2023). Despite the inconsistence between different methods used for RUNX1 inhibition which might be due to the general superiority of cardiotropic AAVs, the findings from Martin et al. (Martin et al. 2023) raised the possibility of limiting infarct size post-MI by antagonizing RUNX1 function which requires further validation, particularly at an earlier stage of cardiac damage. Here, we utilized a small molecule inhibitor (Ro5-3335) as a means of antagonizing RUNX1 and examined its effects on infarct size in the context of acute MI. Adult male Wistar rats were subjected to thoracotomy and left anterior descending coronary artery ligation through approaches we previously established (McCarroll et al. 2018; He et al. 2022). Ro5-3335 was administered immediately following coronary artery ligation by intraperitoneal injection at a dose of 10 mg/kg based on the previous study defining its effects on cardiac contractile function post-MI (Martin et al. 2023). To implement consistent procedures across all animals for the measurement of infarct size, coronary artery ligation was performed at 2.5 mm below the left atrial appendage (Fig. 1A [i] and [ii]). Rats were sacrificed and hearts were collected at 24 hours post-MI followed by immediate TTC staining on fresh heart sections. Infarct size was measured using a previously reported method with minor modifications (Saku et al. 2018). Five transverse slices were taken from the apex to the base of the heart, with a 2 mm distance between slices (Fig. 1A [iii] and [iv]). These slices were stained by TTC and infarct size was measured from the rostral face of each section (Fig. 1B). We report that Ro5-3335-treated rats demonstrated a statistically significant reduction in infarct size by 22% when compared with the vehicle-treated rats (32 ± 2 vs. 25 ± 1% sum of infarct area to the whole LV area; vehicle [*n*=8] vs. Ro5-3335 [*n*=8]; *P*<0.05) (Fig. 1C). Our data demonstrate that infarct size at 1-day post-MI can be reduced by antagonizing RUNX1, the expression of which is known to increase in the BZ post-MI (McCarroll et al. 2018; Martin et al. 2023; Kubin et al. 2011; Gattenlohner et al. 2003).

### Inhibition of RUNX1 leads to proteomic changes in BZ myocardium

**Fig. 2:**
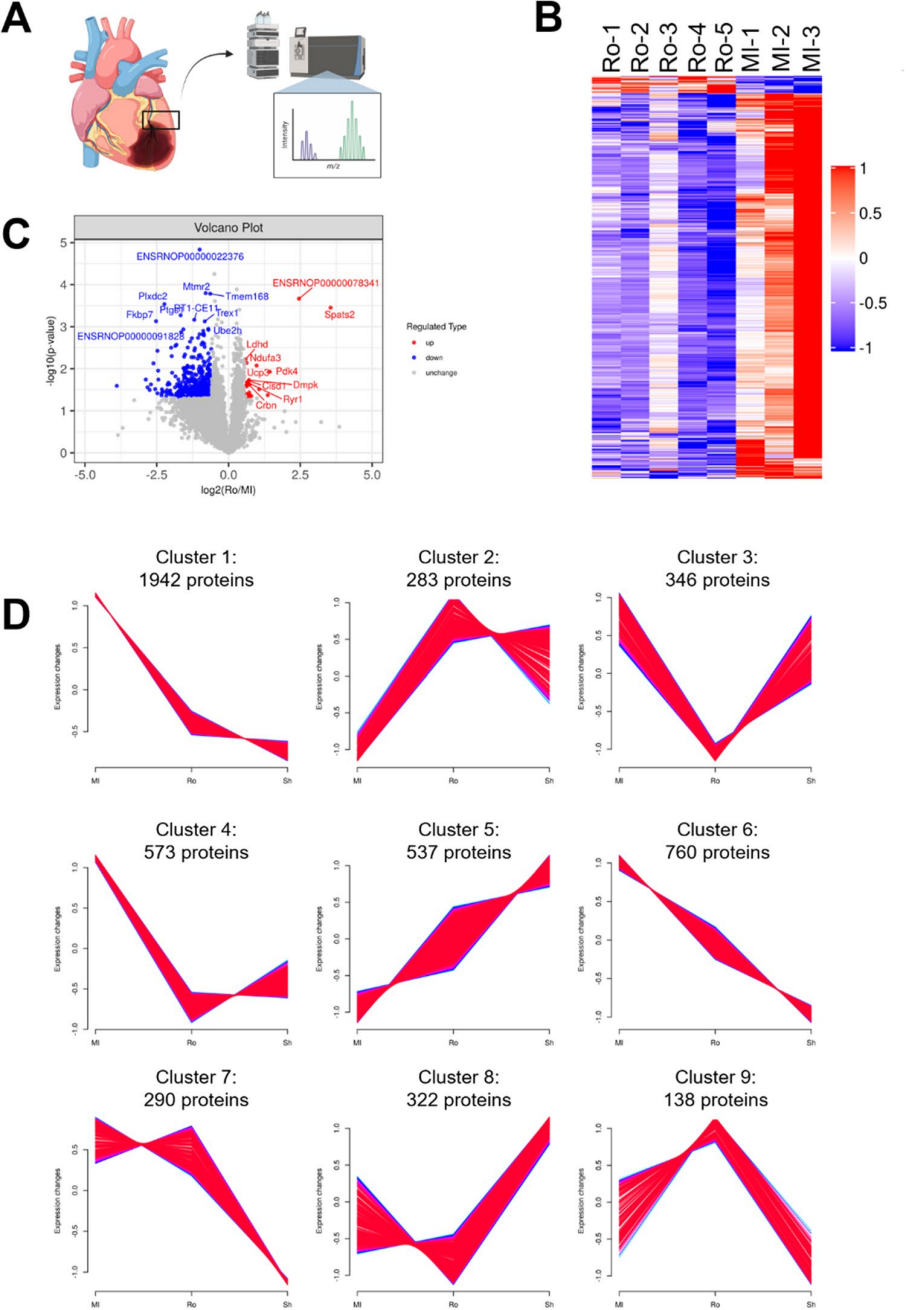
Inhibition of RUNX1 changes protein expression profile. (**A**) Heart tissue samples taken from the BZ at 24 h post-MI was analyzed by proteomics. (**B**) The heatmap (blue to red) represents downregulated or upregulated proteins based on the *Z*-score. See enlarged heatmap in supplementary material. (**C**) The volcano plot shows the distribution of downregulated and upregulated proteins. The x-axis represents the expression of each protein, reported as log2 fold change, while the y-axis represents the −log10 (*P-*value). The red and blue dots highlight the proteins with significant differential expression (fold change>1.5 or <0.67, *P*<0.05). (**D**) Cluster analysis of identified proteins by Mfuzz illustrates 9 clusters with discrete expression changes. Study groups: MI, MI rat hearts treated by vehicle (*n*= 3 hearts); Ro, MI rat hearts treated by Ro5-3335 (*n*= 5 hearts); Sh, sham-operated rat hearts treated by vehicle (*n*= 3 hearts). Figure 2A was created using BioRender.com under a publication license

The BZ is a highly active region post-MI that represents considerable changes in gene expression (Martin et al. 2023), some of which may ultimately drive cell death signaling. The extent to which RUNX1 modulates these changes of gene expression at the protein level within the BZ is unknown. Therefore, we used quantitative proteomics to detect protein expression in the BZ myocardium in vehicle-treated MI rats (MI group, *n*=3 hearts), Ro5-3335-treated MI rats (Ro group, *n*=5 hearts) and vehicle-treated sham-operated rats (Sh group, *n*=3 hearts) at 1-day after MI or sham surgery (Fig. 2A). Heart samples were analyzed by liquid chromatography coupled to tandem mass spectrometry (LC-MS/MS) operating in the data-independent acquisition (DIA) mode. A total of 45257 peptides and 5211 proteins were identified, with a statistical filter based on *Q*-value cutoff 0.01, equivalent to 99% confidence as determined by false discovery rate (FDR<1%). To investigate the role that RUNX1 plays in the BZ, we compared the proteins expressed in the BZ myocardium in the Ro group with that in the MI group. There were 548 differentially expressed proteins between Ro and MI groups (fold change>1.5 or <0.67, *P*<0.05), with 524 proteins downregulated and 24 proteins upregulated in Ro rats relative to MI rats (Fig. 2B and C, see enlarged heatmap in supplementary material). Mfuzz cluster analysis of detected proteins produced 9 clusters with distinct expression patterns (Fig. 2D). Cluster 1 has the largest number of proteins (1942 proteins), showing a trend that RUNX1 inhibition caused decreased expression of a large number of proteins, whereas in the vehicle-treated MI rats these proteins were actively upregulated following MI relative to rats subjected to sham surgery. Cluster 4 including 573 proteins demonstrates a similar pattern with protein expression reduced by RUNX1 inhibitor trending toward lower levels compared with sham group. Proteins clustered in cluster 3 (346 proteins) and cluster 8 (322 proteins) show tendencies of decreased expression by inhibiting RUNX1 but not seem activated in response to MI when comparing MI vehicle-treated MI rats with sham rats. A comparatively small number of proteins in cluster 2 (283 proteins) and cluster 9 (138 proteins) show opposite trends toward upregulation of genes with Ro5-3335 treatment. Altogether, these patterns of expression change suggest that inhibition of RUNX1 leads to the suppression of a considerable number of genes, in agreement with the role of RUNX1 as a master regulatory transcription factor which controls expression of associated genes.

### Subcellular distribution, GO and KEGG enrichment analyses

**Fig. 3:**
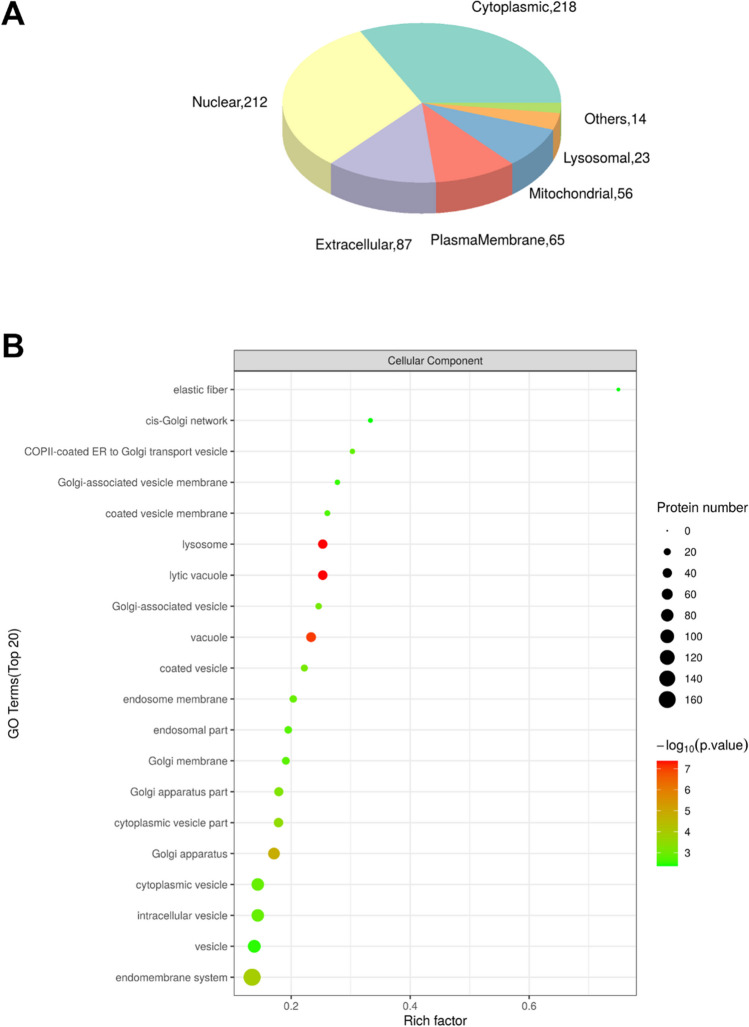
Subcellular and GO enrichment analysis of proteomic data. (**A**) Prediction of subcellular locations of differentially expressed proteins between vehicle- and Ro5-3335-treated MI hearts. (**B**) GO enrichment analysis of differentially expressed proteins in Ro5-3335-treated hearts relative to control MI hearts. X axis shows rich factor which represents the ratio of the number of enriched proteins in a category to the total number of proteins in that category. Y axis shows category names. Area of each node represents the number of enriched proteins differentially expressed upon inhibiting RUNX1. *P*-values are represented by color scale

**Fig. 4:**
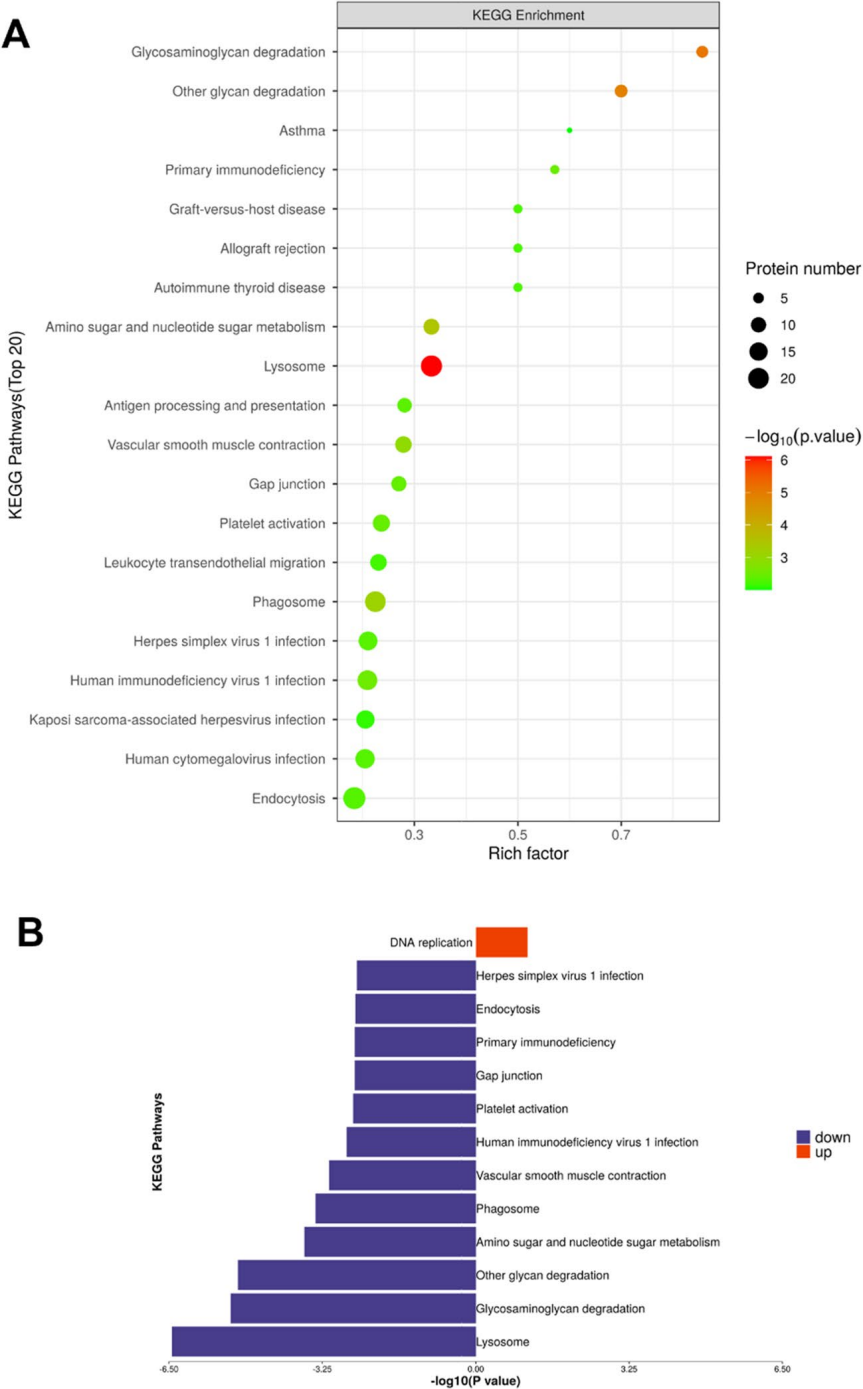
KEGG analysis of proteomic data. (**A**) KEGG enrichment analysis of differentially expressed proteins. X axis shows rich factor which represents the ratio of the number of enriched proteins. Y axis shows category names. Area of each node represents the number of enriched proteins. *P*-values are represented by color scale. (**B**) Top KEGG pathways of differentially expressed proteins upon Ro5-3335-treatment relative to control ranked by enrichment *P*-value. Downregulation and upregulation are represented by blue and red, respectively

Subcellular localization analysis predicted the distribution of differentially expressed proteins in Ro5-3335-treated hearts relative to vehicle-treated MI hearts (Fig. 3A). The subcellular distribution prediction illustrates 218 cytoplasmic proteins, 212 nuclear proteins, 87 extracellular proteins, 65 plasma-membrane proteins, 56 mitochondrial proteins and 23 lysosomal proteins. GO annotation, enrichment and KEGG pathway analyses were performed against proteins that were changed upon RUNX1 inhibition. The top enriched cellular components in GO analysis include several internal membranous organelles associated with lysosomal function, such as lysosome itself, lytic vacuole, endosome, Golgi apparatus, and intracellular vesicle (Fig. 3B). The top enriched pathways in KEGG enrichment analysis involve pathways related to lysosomal function, coagulation and inflammatory responses, such as the lysosome pathway (most significant), phagosome, endocytosis, platelet activation, vascular smooth muscle contraction, and several immune-associated pathways (Fig. 4A). Among these enriched pathways, the top 6 KEGG pathways ranked by *P*-value are lysosome pathway, glycosaminoglycan degradation (which is degraded in lysosomes), amino sugar and nucleotide sugar metabolism, phagosome and vascular smooth muscle contraction (Fig. 4B). Interestingly, most effects obtained by antagonizing RUNX1 implicate downregulation of injury-associated signalings, whilst the pathway of DNA replication demonstrates upregulated function, indicating that inhibition of RUNX1 may cause enhanced proliferation and/or regeneration following MI (Fig. 4B).

### RUNX1 inhibition leads to repressed cathepsin levels

**Fig. 5:**
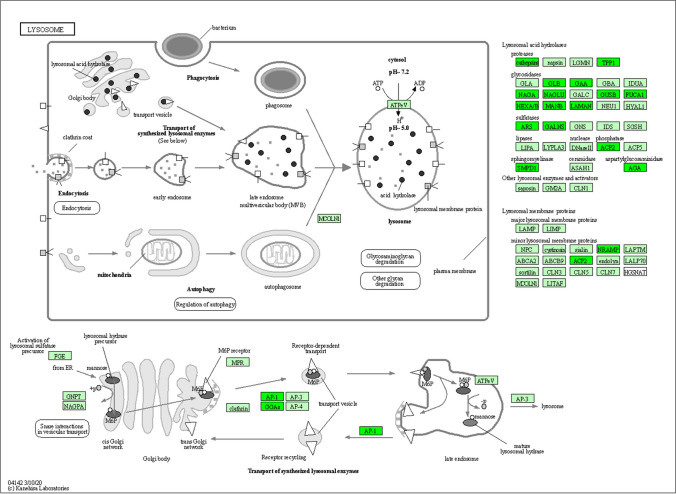
The KEGG lysosome pathway map. Green boxes represent proteins downregulated in the BZ of Ro5-3335-treated hearts in comparison to control MI samples. White boxes indicate unchanged proteins

**Fig. 6:**
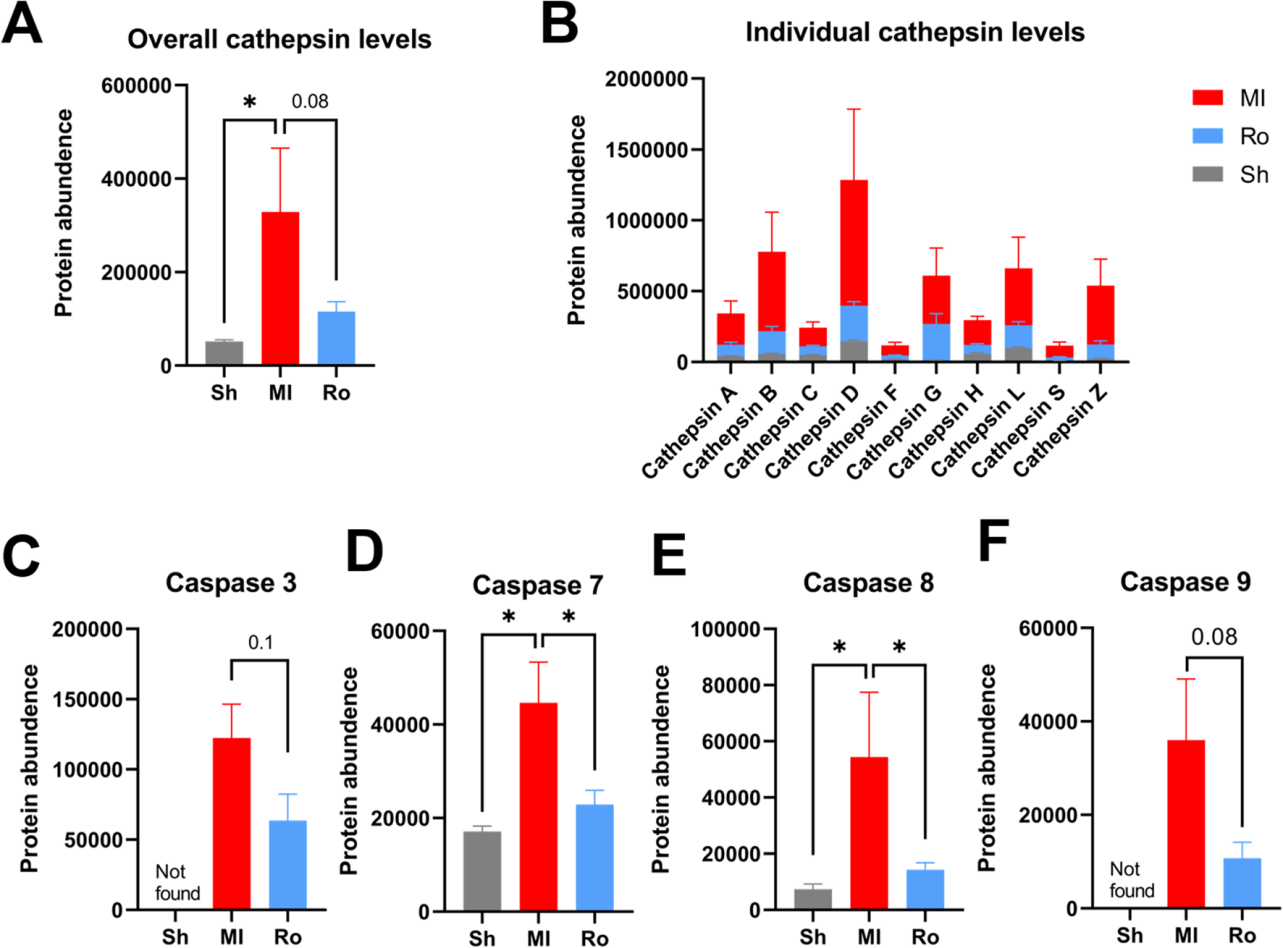
Proteomic quantification of cathepsins and caspases. (**A**) Overall cathepsin levels reflected by the average of identified cathepsin members and protein levels for (**B**) individual cathepsin levels, (**C**) caspase 3, (**D**) caspase 7, (**E**) caspase 8 and (**F**) caspase 9. *P < 0.05. Study groups: MI, MI rat hearts treated by vehicle (*n*=3 hearts); Ro, MI rat hearts treated by Ro5-3335 (*n*= 5 hearts); Sh, sham-operated rat hearts treated by vehicle (*n*=3 hearts)

The results of enrichment analysis indicate that inhibiting RUNX1 function has repressed the expression of lysosomal proteins. The KEGG lysosome pathway map illustrates proteins with decreased expression levels including lysosomal membrane proteins (e.g., LAMP, LIMP), lysosomal hydrolases (e.g., proteases, glycosidases, sulfatases, lipases, nucleases, phosphatases), and proteins associated with transportation of these enzymes (Fig. 5), indicative of decreased lysosomal activity in Ro5-3335-treated infarct hearts. The most abundant proteases that are stored in lysosomal compartments are cathepsins which play a vital role in protein degradation, immune responses, and cell death processes (Yadati et al. 2020; Conus and Simon 2008). Cathepsins are categorized into three groups based on the type of amino acid at the active site: serine cathepsins (cathepsin A and G), aspartic cathepsins (cathepsin D and E) and cysteine cathepsins (cathepsin B, C, F, H, K, L, O, S, V, X, and W) (Yadati et al. 2020; Liu et al. 2018; O'Toole et al. 2020). In our previous study, He et al. (He et al. 2022) demonstrate that inhibition of a specific member of cathepsin (cathepsin-L) leads to improved cardiac function and reduced infarct size in an ex vivo rat model of myocardial ischemia/reperfusion injury. Given the fact that some cathepsin members (such as cathepsin B, D, L, S and K) are known to be involved in the induction of programmed cell death (He et al. 2022; Bhoopathi et al. 2010; de Castro et al. 2016; Nagakannan et al. 2021; Conus and Simon 2008; Xie et al. 2023), it is conceivable that the reduction in infarct size caused by RUNX1 inhibition might be associated with decreased cathepsin levels. To investigate this speculation, we compared the overall cathepsin levels between groups. Overall cathepsin level (analyzed with the average of identified cathepsin members) increased by 6.4-fold in control MI rats relative to sham rats (*P*<0.05; Fig. 6A). In contrast, rats treated by RUNX1 inhibitor demonstrated a numerically, although not statistically, repressed cathepsin level that was 35% of the control MI rats (*P*=0.08; Fig. 5A). Individual cathepsin levels showed similar tendencies as their expression increased in MI hearts but decreased by RUNX1 inhibition (Fig. 6B). The top 5 cathepsin members were cathepsin D, B, L, G and Z as ranked by proteomic quantified abundance (Fig. 6B). Caspase 3, 7, 8 and 9 are markers of apoptotic cell death (Yuan and Ofengeim 2023). Our previous work has shown that inhibition of cathepsin L leads to repressed caspase 3/7 activity and prevents apoptosis in injured cardiomyocytes (He et al. 2022). Here, our proteomic data showed that RUNX1 inhibition tended to normalize the increased expression of caspases (Fig. 6C-F), indicating that decreased extent of apoptosis might be associated with repressed caspase expression. The cleavage of caspases was unable to be detected by the proteomic method we used, and thus the levels of cleaved caspases were unclarified which warrant further investigation.

## Discussion

RUNX1 is a master regulator of transcription factors and a critical regulator of development (Rozen et al. 2023). Such master regulators often appear to act on the top of a regulatory hierarchy that trigger major decisions in developmental processes and are classically known as controlling most of the regulatory activities of other associated transcription factors and genes (Sikdar and Datta 2017; Yang et al. 2018; Imperato et al. 2015; Ichikawa et al. 2004; Ito et al. 2015). In vertebrates, RUNX1 itself is expressed under the spatiotemporal control of two alternative promoters (distal P1 and proximal P2) that lead to the generation of mRNA isoforms differing in their 5′-untranslated region (UTR) and N-terminal coding sequences (Song et al. 2023; Owens et al. 2022). Messenger RNA isoforms may facilitate differential translational regulation in distinct cellular contexts and may influence RUNX1 expression as part of an adaptive response to stress. (Levanon and Groner 2004; Gao et al. 2021; Na et al. 2023). The role of RUNX1 in the heart is an emerging field, with growing evidence showing that RUNX1 is involved in adverse cardiac remodelling following MI. RUNX1 has been known for its expression in neonatal cardiomyocytes, while in adult hearts RUNX1 expression decreases to much lower levels (Kubin et al. 2011; Song et al. 2023; Eulalio et al. 2012). In both patients and animal models with MI, RUNX1 expression is reactivated in the BZ region of the infarct heart (McCarroll et al. 2018; Martin et al. 2023; Kubin et al. 2011; Gattenlohner et al. 2003). Recent studies using experimental MI models showed that tamoxifen-induced Runx1-deficiency preserves myocardial contractility after MI (McCarroll et al. 2018) and inhibiting RUNX1 in the BZ via short-hairpin RNA interference or small molecule Ro5-3335 prevents contractile dysfunction following MI (Martin et al. 2023). As reviewed by Riddell et al. (Riddell et al. 2020), the role of Runx1 in the heart has attracted substantial attention of the cardiovascular field and RUNX1 is thought to be a promising therapeutic target for MI.

The RUNX1 inhibitor Ro5-3335 is a benzodiazepine derivative which is known to suppress RUNX1 activity by inhibiting its transcriptional process (Riddell et al. 2020; Cunningham et al. 2012). Pre-treatment or post-treatment with Ro5-3335 in mice in the context of MI leads to improved cardiac systolic function relative to the vehicle control group (measured by echocardiography). The preserved cardiac function may be partially due to a reduction in infarct size, whereas whether pharmacologically inhibiting RUNX1 affects infarct size was unclear. In the present study, we demonstrate firstly that immediate administration of RUNX1 inhibitor Ro5-3335 following MI reduces infarct size in rats in vivo and the beneficial effects are associated with repressed cardiac cathepsin levels. Notably, although the RUNX1 inhibitor Ro5-3335 is known to inhibit RUNX1 function and this effect is well evidenced (Cunningham et al. 2012; Martin et al. 2023; Delgado-Tirado et al. 2020; Tang et al. 2024; Klase et al. 2014; She et al. 2023; O'Hare et al. 2021), the mechanism of inhibition (whether it’s direct or indirect) is under debate. Whilst Cunningham et al. (Cunningham et al. 2012) reported that the inhibitory effect of Ro5-3335 is direct via bindings to both subunits of the RUNX1 heterodimeric transcription factor complex which is indicated by UV measurement of protein binding, a later study by Illendula et al. (Illendula et al. 2016) pointed out that the effect of Ro5-3335 on RUNX1 function may be indirect because its interaction with the RUNX1 runt domain could not be detected by NMR and suggested that the inhibitory effect of Ro5-3335 may be based on the inhibition of SMARCA2 which is predicted to be an interaction partner of RUNX1 by protein-protein interaction prediction program PIPS (Illendula et al. 2016). Therefore, the precise mechanism of the interaction between Ro5-3335 and RUNX1 is unconfirmed and requires further study.

Recent studies have highlighted the role for RUNX1 in driving cardiac dysfunction under pathological conditions. McCarroll & He et al. (McCarroll et al. 2018) reported firstly that increased RUNX1 expression leads to decreased cardiac contractile function following MI and the underlying mechanism is related to impaired calcium handling. By investigating cardiomyocytes isolated from cardiomyocyte-specific Runx1-deficient mice, this study revealed that calcium transient amplitude was increased and time constant of decline was reduced in Runx1-deficient mice after MI, resulting in an increase in cardiomyocyte shortening (McCarroll et al. 2018). The observed improvement of calcium handling is largely attributed to enhanced sarcoplasmic reticulum (SR)-mediated calcium uptake via SERCA in the Runx1-deficient mice. Although expression of phospholamban is not altered, phosphorylation of phospholamban activates SERCA function in Runx1-deficient mice after MI, leading to an increased SR calcium content, which in turn increases electrically induced SR-mediated calcium release and improves cell contraction (McCarroll et al. 2018). Martin et al. (Martin et al. 2023) demonstrated that antagonizing RUNX1 expression genetically by short-hairpin RNA interference preserved contractile function after MI. Beneficial effects on cardiac function were equivalently obtained with pharmacological inhibition using Ro5-3335 (Martin et al. 2023). These findings suggest that RUNX1 can be therapeutically targeted to preserve cardiac contractile function after MI. It is important to recognize that RUNX1 also has a critical role in non-cardiomyocyte cells. More recently, Amrute et al. (Amrute et al. 2023) performed single-nucleus RNA sequencing from patients with heart failure and found that downregulation of Runx1 may modulate macrophage phenotype. Intriguingly, a linear regression for LV ejection fraction versus Runx1 expression quantified via pseudobulk analyses at the patient level revealed a strong negative correlation (Amrute et al. 2023). Runx1 expression was also observed in cardiac fibroblasts along with genes contributing to myocardial fibrosis, such as POSTN, MEOX1 and FAP, and its expression was also negatively correlated to ejection fraction (Amrute et al. 2023). The negative correlation between Runx1 expression and heart function shown in the above study is consistent with our previous study where Runx1-deficient mice exhibited preserved contractile function and were protected against heart failure following MI (McCarroll et al. 2018). Compared to these previous studies which demonstrated that RUNX1 is associated with cardiac dysfunction, the present study is focused on its impact on infarct size and related mechanisms.

Cathepsins are a key set of enzymes that present in lysosomes which are also known as lysosomal carboxypeptidases (Naqvi et al. 2022). Lysosome-dependent cell death is one of the subroutines of regulated cell death identified by the Nomenclature Committee on Cell Death (Galluzzi et al. 2018) and is characterized by the permeabilization of lysosomal membrane followed by the release of lysosomal cathepsins into cytosol (Tang et al. 2019; Luke et al. 2022). However, there have been difficulties in ascertaining lysosome-dependent cell death in that lysosomal membrane permeabilization and cathepsin release can also be found in other cell death subroutines including apoptosis, mitochondrial permeability transition-driven necrosis, pyroptosis, ferroptosis, and necroptosis (Luke et al. 2022). Indeed, the role of cathepsins in mediating multiple forms of regulated cell death is well documented (Conus and Simon 2008; Bhoopathi et al. 2010; Nagakannan et al. 2021; Xie et al. 2023; Luke et al. 2022; Ding et al. 2022), regardless of which subroutines they involve. The high-throughput proteomics based on mass spectrometry allows characterization of the cathepsin family members in tissue samples which facilitates our investigation of the whole cathepsin family. By analyzing heart samples taken from our well-established murine MI models (McCarroll et al. 2018; He et al. 2022; Martin et al. 2022), we have detected 10 cathepsin members (cathepsin A, B, C, D, F, G, H, L, S, Z) with DIA-based quantitative proteomics and found the expression of these cathepsins all trended toward upregulation in the BZ following MI and inhibition of RUNX1 tended to normalize their expression levels.

The top 5 cathepsin members overexpressed in the BZ of the MI heart ranked by our proteomic quantification are cathepsin D, B, L, G and Z. The roles of these cathepsins in mediating cell death signalings are well evidenced through a variety of studies. Deiss et al. (Deiss et al. 1996) showed that ectopic expression of cathepsin D induces cell death in the absence of any external stimulus. Moreover, overexpression of cathepsin D was observed during cell death, with high levels of proteolytical activity. By contrast, the cathepsin D antisense RNA and cathepsin D inhibitor (Pepstatin A) suppressed cell death in HeLa cells. Zuzarte-Luis et al. (Zuzarte-Luis et al. 2007) showed upregulated expression of cathepsin D in embryo tissues undergoing apoptosis during development and demonstrated a reliable technique to outline the areas of cell death by detecting cathepsin D expression. Cathepsin B is currently the best-characterized member of lysosomal cysteine cathepsins and the leakage of cathepsin B from lysosome is thought to be a trigger of the pathological development of various diseases. A recent review by Xie et al. (Xie et al. 2023) highlighted the role that cathepsin B plays in multiple types of programmed cell death. Cellular intrinsic damage causes lysosomal cathepsin B leakage which mediates the degradation of anti-apoptotic B-cell lymphoma (Bcl)-2 family proteins. Pro-apoptotic proteins, such as Bcl-2-like protein 4 (Bax) and Bcl-2 antagonist/killer 1 (Bak), tend to induce mitochondrial outer membrane permeabilization, where the anti-apoptotic Bcl-2 is sequestered by cathepsin B (Heimer et al. 2019). Pro-apoptotic factors are subsequently released from the mitochondria, forming the so-called apoptosome complex which allows autoactivation of caspase-9 followed by the activation of the apoptotic executioner caspase-3/7, thereby prompting apoptosis (Elena-Real et al. 2018). Importantly, serum levels of cathepsin L are elevated among patients with coronary heart disease (Zhang et al. 2010; Liu et al. 2009) and the cardiac release of cathepsin L is associated with apoptosis. In our previous study, He et al. (He et al. 2022) showed that inhibition of cathepsin L following ischemia/reperfusion injury reduces infarct size and improves cardiac contractile function by improving cardiomyocyte function and preventing apoptosis. Cathepsin G demonstrates biological functions related to programmed necrosis (Chaitanya et al. 2010). Burgener et al. (Burgener et al. 2019) showed that inhibition of cathepsin G by intracellular protease inhibitors Serpinb1a and Serpinb6a limits multiple forms of cell death in neutrophils and monocytes. Cathepsin Z is involved in inflammation and IL-1β production (Campden et al. 2022), and it is also known to promote apoptosis in neuronal cells (Pišlar and Kos 2014).

**Fig. 7:**
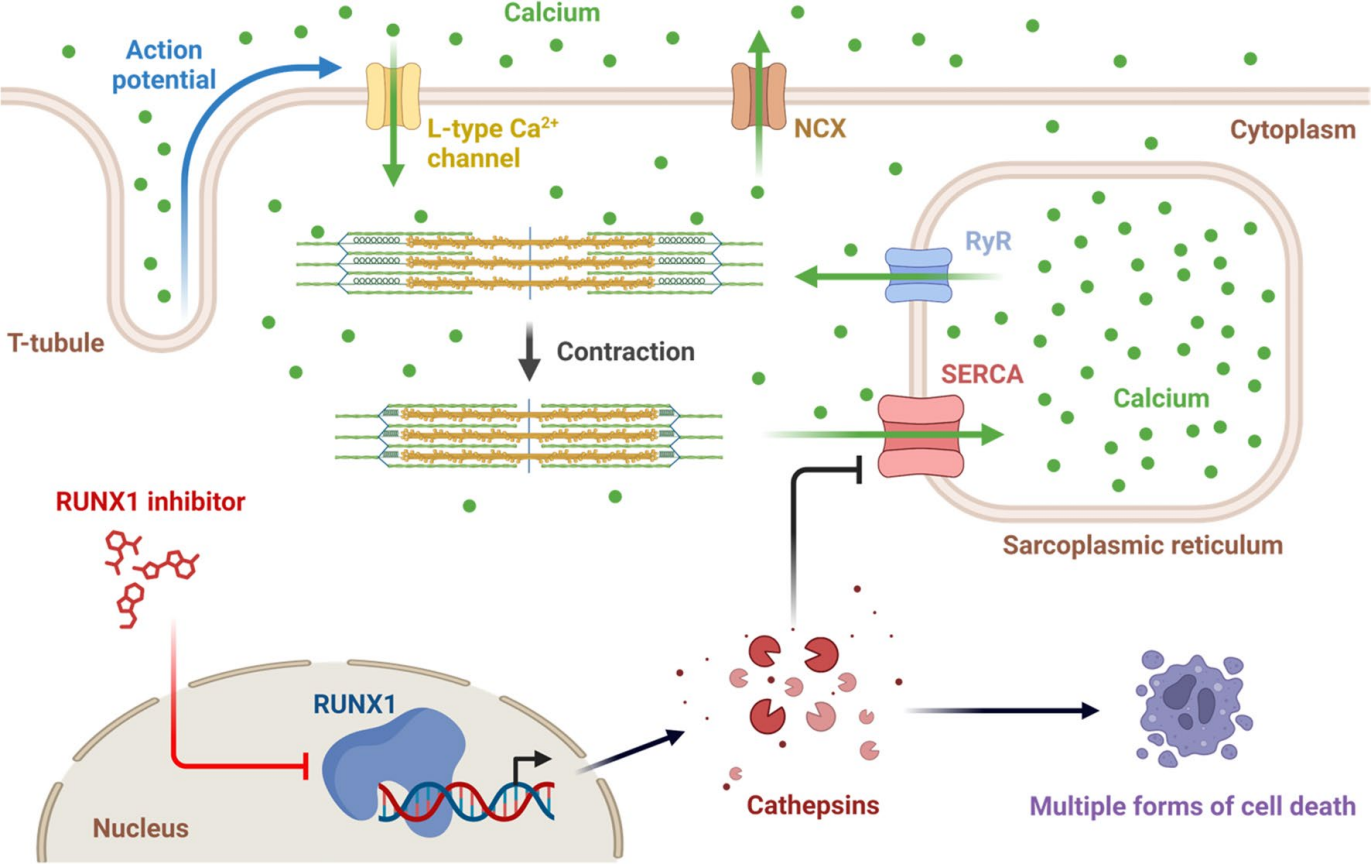
Schematic depicting roles of RUNX1 and cathepsins in cardiomyocytes following myocardial infarction or ischemia/reperfusion injury. RUNX1 is a master-regulator transcription factor which controls gene expression. Cathepsins seem to be regulated by RUNX1 and act as executioners to mediate contractile dysfunction and various forms of cell death. NCX, sodium-calcium exchanger; RyR, ryanodine receptor calcium release channels; SERCA, sarco/endoplasmic reticulum Ca^2+^-ATPase. Figure 7 was created using BioRender.com under a publication license, based on information from this study and various sources (McCarroll et al. 2018; He et al. 2022; Yadati et al. 2020; Nagakannan et al. 2021; Xie et al. 2023)

Infarct size is a major determinant of prognosis and the target of cardioprotective therapies in patients with MI (Heusch 2020). The present study demonstrates that inhibition of RUNX1 following MI with Ro5-3335 reduces infarct size and decreases cathepsin levels. Multiple lines of evidence suggest that the beneficial effects on infarct size obtained by inhibiting RUNX1 are associated with repressed cathepsin levels: (1) RUNX1 expression is increased in the BZ and IZ regions of the heart at an early-stage following MI and mediates impaired cardiac function (McCarroll et al. 2018; Martin et al. 2023; Kubin et al. 2011; Gattenlohner et al. 2003). In the present study, cathepsins show similar trends of change in their expression levels for 10 members of cathepsins detected in the BZ tissue by quantitative proteomics. (2) There has been convincing evidence that cathepsin family members are involved in multiple cell death pathways as executioners and modulators (Nagakannan et al. 2021; Conus and Simon 2008; Xie et al. 2023; Luke et al. 2022; Turk and Stoka 2007). Our prior study also confirms that inhibition of a specific cathepsin member (cathepsin-L) leads to reduced infarct size (He et al. 2022), suggestive of an underlying mechanism that inhibition of RUNX1 may reduce infarct size via suppressing cathepsin-mediated cell death. (3) Considering the function of RUNX1 as a master-regulator transcription factor that controls gene expression either directly through transcriptional modulation, or indirectly through interactions with other transcription factors (e.g., PU.1, CEBPA, GATA1) (Riddell et al. 2020; Imperato et al. 2015; Xu et al. 2006; Elagib et al. 2003; Grossmann et al. 2012; Guo et al. 2012), it is likely that the expression of cathepsins is under the regulation of RUNX1, at least to some extent. (4) Our previous data have shown similar changes in calcium handling in both Runx1-deficient cardiomyocytes and cardiomyocytes treated by cathepsin-L inhibitor. McCarroll & He et al. (McCarroll et al. 2018) demonstrated enhanced sarco/endoplasmic reticulum Ca^2+^-ATPase (SERCA) activity in Runx1-deficient mice following MI, with increased sarcoplasmic reticulum calcium content and increased sarcoplasmic reticulum–mediated calcium release. He et al. (He et al. 2022) showed that in cardiomyocytes isolated from ex vivo rat hearts subjected to ischemia/reperfusion injury, inhibition of cathepsin-L resulted in an increased calcium transient amplitude, enhanced SERCA activity and improved NCX activity (similar to the effects observed in the Runx1-deficient mice). Since calcium dysregulation is known to cause mitochondrial failure and cell death during ischemia/reperfusion injury or following MI (Garcia-Dorado et al. 2012), the reduction in infarct size observed by the present work may also be partly attributed to improved calcium handling (Fig. 7).

This study has some limitations. First, this work only measured infarct size at a single time-point and lacked data at later time-point reflecting the fate of cardiac tissue. Since previous study has shown that genetic inhibition of Runx1 led to reduced infarct size and improved cardiac function at 7-days post-MI (Martin et al. 2023). Our study suggests that these beneficial effects may be associated with a smaller infarct size obtained through Runx1 inhibition at the acute phase of injury. The reduction of infarct size at an early time-point has clinical significance because the time of intervention is within the therapeutic window and pharmacological inhibition of RUNX1 has the potential to be translated into clinical application. Second, the informative proteomics data reflect a consistent trend of changes of the whole cathepsin family. We recognize that these findings have not been corroborated by other experimental approaches, such as western blot, cathepsin activities, and cathepsin subtype discrimination, which would strengthen the possible mechanism suggested by this work. Third, whilst the main focus of this study is on infarct size which was statistically significant between groups, the proteomic data which provide mechanistic evidence to the observed reduction in infarct size were not adequately powered to show statistical differences between Ro and MI groups. Based on the fact that cathepsins mediate cell death signaling is well-evident, our findings suggests that the observed reduced infarct size may be related to repressed cathepsin levels. Moreover, there is a possibility that the differential effects between pharmacological and transcriptional inhibition may depend on the levels of inhibition achieved. We also recognize that the RUNX1 inhibitor Ro5-3335 may have side effects and additional way of actions. Thus, a comparison of pharmacological inhibition of RUNX1 to that mediated by shRNA would be necessary to address this issue in future studies. Finally, we have not investigated the underlying mechanism of the association between RUNX1 inhibition and decreased cathepsin levels. Further study is merited to explore the mechanism by which RUNX1 inhibition reduces cathepsin expression.

## Conclusion

Taken together, this study provides additional insight into the role of RUNX1 in the pathological cell death processes following MI. In an in vivo rat model of acute MI, small molecule inhibition of RUNX1 has shown beneficial effects, reducing infarct size and decreasing cathepsin levels. Our findings suggest a new link between RUNX1 and the cathepsin-mediated cell death which has significant implications for cardiac protection and will probably initiate further research into pharmacologically targeting RUNX1 in an alternative clinically relevant surgical model. In this model, the blocked coronary artery is subsequently opened following a period of ischemia (He et al. 2022), as would be the case for treating patients with percutaneous coronary intervention, further supporting the translational potential of RUNX1. We envisage that using RUNX1 as a multi-targeted approach could mitigate disease progression among patients with MI, thereby improving survival rates and quality of life.

## Methods

### Animal husbandry

Adult male Wistar rats weighing 250-300 g were purchased from Chengdu Dashuo Experimental Animal Co., Ltd. and were randomly assigned to experimental groups. Animals were housed in standard environmental conditions (20-22°C and 12 h light-dark cycle), fed with standard food (Formula Diet for rodents; Dashuo, Chengdu, China) and high purity water. All animal procedures were approved by the Sichuan University Medical Ethics Committee. The care and use of animals were in accordance with local and international ethical criteria.

### Coronary artery ligation

Rats were initially anaesthetized by 4% isoflurane (RWD, China) receiving oxygen at 1 L/min. Rats received preoperative analgesia of 5 mg/kg carprofen and 0.1 mg/kg buprenorphine delivered subcutaneously in a single injection made up with 1 mL saline. Rats were endotracheally intubated and mechanically ventilated at 80 breath/min with a tidal volume of 1.2 ml per 100 g body weight. Isoflurane was reduced to 3.5% initially and then gradually reduced to 1% throughout the surgery. A 3 cm skin incision was made across the rib cage and thoracic muscles were retracted by a chest retractor. The intercostal muscles were blunt dissected and the pericardium was opened to expose the heart. The left anterior descending (LAD) coronary artery was ligated with a 6-0 non-absorbable prolene suture (Ethilon; Johnson & Johnson, China) 2.5 mm distal to the left atrium, with successful ligation confirmed by visualization of myocardial blanching distal to the suture and dysfunction of the ischemic area. The lungs were reinflated and the ribs were drawn together using 2-0 non-absorbable prolene sutures (Ethilon; Johnson & Johnson, China) with an interrupted suture pattern. The thoracic muscles were returned to their original positions and the skin was closed using 6-0 absorbable vicryl (Ethicon, Johnson & Johnson, China) with a continuous suture pattern. Two rats died during surgery due to respiratory failure and were excluded from analysis. Other animals included in this study were all survived from the surgical procedure.

### Infarct size

Rats were anaesthetized in a pre-filled induction chamber with 4% isoflurane in 1.0 L/min oxygen and then killed by cervical dislocation. Each heart was cut into 5 horizontal slices from the apex to base, with a 2 mm distance between slices. Heart slices were stained with 2% triphenyltetrazolium chloride (Shanghai yuanye Bio-Technology, China) at 37°C for 15 min. These stained slices were photographed against the rostral face and infarct size was quantified as the ratio of the sum of infarct area in 5 slices to the sum of whole LV area in the same 5 slices using ImageJ.

### Mass spectrometry-based proteomics

Rats were anaesthetized with 4% isoflurane in 1.0 L/min oxygen and then killed by cervical dislocation. Heart tissue samples from border zone in MI hearts and from the corresponding region in sham hearts were carefully dissected and collected for proteomic analysis. Tissue samples were homogenized by MP FastPrep-24 homogenizer (6.0 M/S, 60 s, twice) and SDT buffer (4% SDS, 100 mM DTT, 150 mM Tris-HCl pH 8.0) was added. The lysates were sonicated, boiled for 15 min, and centrifuged at 14000g for 40 min. The supernatant was quantified with the BCA Protein Assay Kit (Bio-Rad, USA) and stored at -80°C. The samples were analyzed by LC-MS/MS operating in the DIA mode at the Shanghai Applied Protein Technology Co., Ltd. Each DIA cycle contained one full MS–SIM scan, and 30 DIA scans covered a mass range of 350–1800 m/z. QC samples monitoring the MS performance (pooled sample from equal aliquot of each sample) were injected at the beginning of the MS study and after every 6 injections throughout the experiment. Spectronaut^TM^ (Biognosys) searching was performed at the Shanghai Applied Protein Technology Co.,Ltd., with constructed spectral library. All results were filtered based on *Q*-value cutoff 0.01 (equivalent to FDR<1%)

### Bioinformatic analysis

Clustering of protein expression data was performed with Mfuzz. Prediction of protein subcellular localization was carried out with a multi-class classification system, CELLO (http://cello.life.nctu.edu.tw/). Homology searching was performed using NCBI BLAST and InterProScan against the differentially expressed proteins. Gene ontology (GO) terms were mapped and sequences were annotated using the Blast2GO software. Following annotation, proteins were blasted using the online Kyoto Encyclopedia of Genes and Genomes (KEGG) database (http://geneontology.org/). The retrieved KEGG orthology identifications were subsequently mapped to pathways. Enrichment analysis was performed with Fisher’s exact test and the whole quantified proteins were used as background dataset. Benjamini-Hochberg correction for multiple testing was used to adjust *P*-values. Functional categories and pathways with *P*-values under 0.05 were considered significant. The enrichment results were plotted by R scripts.

### Statistics

For statistical significance of differences between groups, raw data were analyzed with GraphPad Prism and are presented as mean ± standard error of the mean (SEM). Statistical comparisons were made by a two-sample Student’s T-test. Multiple groups were compared with analysis of 1-way variance (ANOVA) followed by Dunnett’s post-hoc test. The *P*-values below a threshold of 0.05 were considered statistically significant.

### Supplementary information

ESM 1 (JPG 5071 kb)

## Data Availability

The datasets used and/or analyzed during the current study are available from the corresponding author on reasonable request.

## References

[CR1] Amrute JM, Lai L, Ma P, Koenig AL, Kamimoto K, Bredemeyer A, Shankar TS, Kuppe C, Kadyrov FF, Schulte LJ (2023). Defining cardiac functional recovery in end-stage heart failure at single-cell resolution. Nat Cardiovasc Res.

[CR2] Bhoopathi P, Chetty C, Gujrati M, Dinh DH, Rao JS, Lakka S (2010). Cathepsin B facilitates autophagy-mediated apoptosis in SPARC overexpressed primitive neuroectodermal tumor cells. Cell Death Differ.

[CR3] Bulluck H, Yellon DM, Hausenloy DJ (2016). Reducing myocardial infarct size: challenges and future opportunities. Heart (British Cardiac Society).

[CR4] Burgener SS, Leborgne NGF, Snipas SJ, Salvesen GS, Bird PI, Benarafa C (2019). Cathepsin G Inhibition by Serpinb1 and Serpinb6 Prevents Programmed Necrosis in Neutrophils and Monocytes and Reduces GSDMD-Driven Inflammation. Cell Rep.

[CR5] Campden RI, Warren AL, Greene CJ, Chiriboga JA, Arnold CR, Aggarwal D, McKenna N, Sandall CF, MacDonald JA, Yates RM (2022). Extracellular cathepsin Z signals through the α(5) integrin and augments NLRP3 inflammasome activation. J Biol Chem.

[CR6] Chaitanya GV, Alexander JS, Babu PP (2010). PARP-1 cleavage fragments: signatures of cell-death proteases in neurodegeneration. Cell Commun Signal.

[CR7] Chareonthaitawee P, Christian TF, Hirose K, Gibbons RJ, Rumberger JA (1995). Relation of initial infarct size to extent of left ventricular remodeling in the year after acute myocardial infarction. J Am Coll Cardiol.

[CR8] Conus S, Simon HU (2008). Cathepsins: key modulators of cell death and inflammatory responses. Biochem Pharmacol.

[CR9] Cunningham L, Finckbeiner S, Hyde RK, Southall N, Marugan J, Yedavalli VR, Dehdashti SJ, Reinhold WC, Alemu L, Zhao L (2012). Identification of benzodiazepine Ro5-3335 as an inhibitor of CBF leukemia through quantitative high throughput screen against RUNX1-CBFbeta interaction. Proc Natl Acad Sci U S A.

[CR10] de Castro MAG, Bunt G, Wouters FS (2016). Cathepsin B launches an apoptotic exit effort upon cell death-associated disruption of lysosomes. Cell Death Discov.

[CR11] Deiss LP, Galinka H, Berissi H, Cohen O, Kimchi A (1996). Cathepsin D protease mediates programmed cell death induced by interferon-gamma, Fas/APO-1 and TNF-alpha. The EMBO J.

[CR12] Del Buono MG, Montone RA, Rinaldi R, Gurgoglione FL, Meucci MC, Camilli M, Iannaccone G, Sanna T, Pedicino D, Trani C (2021). Clinical predictors and prognostic role of high Killip class in patients with a first episode of anterior ST-segment elevation acute myocardial infarction. J Cardiovasc Med.

[CR13] Del Buono MG, Moroni F, Montone RA, Azzalini L, Sanna T, Abbate A (2022). Ischemic Cardiomyopathy and Heart Failure After Acute Myocardial Infarction. Curr Cardiol Rep.

[CR14] Delgado-Tirado S, Amarnani D, Zhao G, Rossin EJ, Eliott D, Miller JB, Greene WA, Ramos L, Arevalo-Alquichire S, Leyton-Cifuentes D (2020). Topical delivery of a small molecule RUNX1 transcription factor inhibitor for the treatment of proliferative vitreoretinopathy. Sci Rep.

[CR15] Ding X, Zhang C, Chen H, Ren M, Liu X (2022). Cathepsins Trigger Cell Death and Regulate Radioresistance in Glioblastoma. Cells.

[CR16] Elagib KE, Racke FK, Mogass M, Khetawat R, Delehanty LL, Goldfarb AN (2003). RUNX1 and GATA-1 coexpression and cooperation in megakaryocytic differentiation. Blood.

[CR17] Elena-Real CA, Díaz-Quintana A, González-Arzola K, Velázquez-Campoy A, Orzáez M, López-Rivas A, Gil-Caballero S, De la Rosa M, Díaz-Moreno I (2018). Cytochrome c speeds up caspase cascade activation by blocking 14-3-3ε-dependent Apaf-1 inhibition. Cell Death Dis.

[CR18] Eulalio A, Mano M, Dal Ferro M, Zentilin L, Sinagra G, Zacchigna S, Giacca M (2012). Functional screening identifies miRNAs inducing cardiac regeneration. Nature.

[CR19] Galluzzi L, Vitale I, Aaronson SA, Abrams JM, Adam D, Agostinis P, Alnemri ES, Altucci L, Amelio I, Andrews DW (2018). Molecular mechanisms of cell death: recommendations of the Nomenclature Committee on Cell Death 2018. Cell Death Differ.

[CR20] Gao K, Zhang F, Chen K, Li W, Guan Y-B, Xu M-L, Chong T, Dai Z-M (2021). Expression patterns and prognostic value of RUNX genes in kidney cancer. Sci Rep.

[CR21] Garcia-Dorado D, Ruiz-Meana M, Inserte J, Rodriguez-Sinovas A, Piper HM (2012). Calcium-mediated cell death during myocardial reperfusion. Cardiovasc Res.

[CR22] Gattenlohner S, Waller C, Ertl G, Bultmann BD, Muller-Hermelink HK, Marx A (2003). NCAM(CD56) and RUNX1(AML1) are up-regulated in human ischemic cardiomyopathy and a rat model of chronic cardiac ischemia. Am J Pathol.

[CR23] Grossmann V, Bacher U, Kohlmann A, Butschalowski K, Roller A, Jeromin S, Dicker F, Kern W, Schnittger S, Haferlach T (2012). Expression of CEBPA is reduced in RUNX1-mutated acute myeloid leukemia. Blood Cancer J.

[CR24] Guo H, Ma O, Speck NA, Friedman AD (2012). Runx1 deletion or dominant inhibition reduces Cebpa transcription via conserved promoter and distal enhancer sites to favor monopoiesis over granulopoiesis. Blood.

[CR25] He W, McCarroll CS, Nather K, Ford K, Mangion K, Riddell A, O'Toole D, Zaeri A, Corcoran D, Carrick D (2022). Inhibition of myocardial cathepsin-L release during reperfusion following myocardial infarction improves cardiac function and reduces infarct size. Cardiovasc Res.

[CR26] Heimer S, Knoll G, Schulze-Osthoff K, Ehrenschwender M (2019). Raptinal bypasses BAX, BAK, and BOK for mitochondrial outer membrane permeabilization and intrinsic apoptosis. Cell Death Dis.

[CR27] Heusch G (2020). Myocardial ischaemia–reperfusion injury and cardioprotection in perspective. Nat Rev Cardiol.

[CR28] Ichikawa M, Asai T, Chiba S, Kurokawa M, Ogawa S (2004). Runx1/AML-1 Ranks as a Master Regulator of Adult Hematopoiesis. Cell Cycle.

[CR29] Illendula A, Gilmour J, Grembecka J, Tirumala VSS, Boulton A, Kuntimaddi A, Schmidt C, Wang L, Pulikkan JA, Zong H (2016). Small Molecule Inhibitor of CBFβ-RUNX Binding for RUNX Transcription Factor Driven Cancers. EBioMedicine.

[CR30] Imperato MR, Cauchy P, Obier N, Bonifer C (2015). The RUNX1–PU.1 axis in the control of hematopoiesis. Int J Hematol.

[CR31] Ito Y, Bae S-C, Chuang LSH (2015). The RUNX family: developmental regulators in cancer. Nat Rev Cancer.

[CR32] Jackson BM, Gorman JH, Moainie SL, Guy TS, Narula N, Narula J, John-Sutton MG, Edmunds LH, Gorman RC (2002). Extension of borderzone myocardium in postinfarction dilated cardiomyopathy. J Am Coll Cardiol.

[CR33] Kehat I, Molkentin JD (2010). Molecular pathways underlying cardiac remodeling during pathophysiological stimulation. Circulation.

[CR34] Klase Z, Yedavalli VS, Houzet L, Perkins M, Maldarelli F, Brenchley J, Strebel K, Liu P, Jeang KT (2014). Activation of HIV-1 from latent infection via synergy of RUNX1 inhibitor Ro5-3335 and SAHA. PLoS Pathogens.

[CR35] Konstam MA, Kramer DG, Patel AR, Maron MS, Udelson JE (2011). Left ventricular remodeling in heart failure: current concepts in clinical significance and assessment. JACC Cardiovasc Imaging.

[CR36] Krishnan V (2023). The RUNX Family of Proteins, DNA Repair, and Cancer. Cells.

[CR37] Kubin T, Poling J, Kostin S, Gajawada P, Hein S, Rees W, Wietelmann A, Tanaka M, Lorchner H, Schimanski S (2011). Oncostatin M is a major mediator of cardiomyocyte dedifferentiation and remodeling. Cell Stem Cell.

[CR38] Levanon D, Groner Y (2004). Structure and regulated expression of mammalian RUNX genes. Oncogene.

[CR39] Lindsey ML, Bolli R, Canty JM, Du XJ, Frangogiannis NG, Frantz S, Gourdie RG, Holmes JW, Jones SP, Kloner RA (2018). Guidelines for experimental models of myocardial ischemia and infarction. Am J Physiol Heart Circ Physiol.

[CR40] Liu CL, Guo J, Zhang X, Sukhova GK, Libby P, Shi GP (2018). Cysteine protease cathepsins in cardiovascular disease: from basic research to clinical trials. Nat Rev Cardiol.

[CR41] Liu Y, Li X, Peng D, Tan Z, Liu H, Qing Y, Xue Y, Shi G-P (2009). Usefulness of Serum Cathepsin L as an Independent Biomarker in Patients With Coronary Heart Disease. Am J Cardiol.

[CR42] Luke CJ, Markovina S, Good M, Wight IE, Thomas BJ, Linneman JM, Lanik WE, Koroleva O, Coffman MR, Miedel MT (2022). Lysoptosis is an evolutionarily conserved cell death pathway moderated by intracellular serpins. Commun Biol.

[CR43] Martin TP, MacDonald EA, Bradley A, Watson H, Saxena P, Rog-Zielinska EA, Raheem A, Fisher S, Elbassioni AAM, Almuzaini O (2023). Ribonucleicacid interference or small molecule inhibition of Runx1 in the border zone prevents cardiac contractile dysfunction following myocardial infarction. Cardiovasc Res.

[CR44] Martin TP, MacDonald EA, Elbassioni AAM, O'Toole D, Zaeri AAI, Nicklin SA, Gray GA, Loughrey CM (2022). Preclinical models of myocardial infarction: from mechanism to translation. Br J Pharmacol.

[CR45] McCarroll CS, He W, Foote K, Bradley A, McGlynn K, Vidler F, Nixon C, Nather K, Fattah C, Riddell A (2018). Runx1 Deficiency Protects Against Adverse Cardiac Remodeling After Myocardial Infarction. Circulation.

[CR46] Mevel R, Draper JE, Lie ALM, Kouskoff V, Lacaud G (2019). RUNX transcription factors: orchestrators of development. Development.

[CR47] Na Y, Hall A, Yu Y, Hu L, Choi K, Burgard JA, Szabo S, Huang G, Ratner N, Wu J (2023). Runx1/3-driven adaptive endoplasmic reticulum stress pathways contribute to neurofibromagenesis. Oncogene.

[CR48] Nagakannan P, Islam MI, Conrad M, Eftekharpour E (2021). Cathepsin B is an executioner of ferroptosis. Biochim Biophys Acta Mol Cell Res.

[CR49] Naqvi SAR, Sherazi TA, Shahzad SA, Javed MR, Nadeem S, Imran M, Rasheed R, Hamid Akash MS, Rehman K (2022). Chapter 8 - Biochemistry of cathepsins enzymes and their metabolic activity in the lysosome. Biochemistry of Drug Metabolizing Enzymes.

[CR50] O'Hare M, Amarnani D, Whitmore HAB, An M, Marino C, Ramos L, Delgado-Tirado S, Hu X, Chmielewska N, Chandrahas A (2021). Targeting Runt-Related Transcription Factor 1 Prevents Pulmonary Fibrosis and Reduces Expression of Severe Acute Respiratory Syndrome Coronavirus 2 Host Mediators. Am J Pathol.

[CR51] O'Toole D, Zaeri AAI, Nicklin SA, French AT, Loughrey CM, Martin TP (2020). Signalling pathways linking cysteine cathepsins to adverse cardiac remodelling. Cell Signal.

[CR52] Owens DDG, Anselmi G, Oudelaar AM, Downes DJ, Cavallo A, Harman JR, Schwessinger R, Bucakci A, Greder L, de Ornellas S (2022). Dynamic Runx1 chromatin boundaries affect gene expression in hematopoietic development. Nat Commun.

[CR53] Pišlar A, Kos J (2014). Cysteine cathepsins in neurological disorders. Mol Neurobiol.

[CR54] Riddell A, McBride M, Braun T, Nicklin SA, Cameron E, Loughrey CM, Martin TP (2020). RUNX1: an emerging therapeutic target for cardiovascular disease. Cardiovasc Res.

[CR55] Rozen EJ, Ozeroff CD, Allen MA (2023). RUN(X) out of blood: emerging RUNX1 functions beyond hematopoiesis and links to Down syndrome. Human Genom.

[CR56] Saku K, Kakino T, Arimura T, Sunagawa G, Nishikawa T, Sakamoto T, Kishi T, Tsutsui H, Sunagawa K (2018). Left Ventricular Mechanical Unloading by Total Support of Impella in Myocardial Infarction Reduces Infarct Size, Preserves Left Ventricular Function, and Prevents Subsequent Heart Failure in Dogs. Circ Heart Fail.

[CR57] Savarese G, Becher PM, Lund LH, Seferovic P, Rosano GMC, Coats AJS (2023). Global burden of heart failure: a comprehensive and updated review of epidemiology. Cardiovasc Res.

[CR58] She C, Wu C, Guo W, Xie Y, Li S, Liu W, Xu C, Li H, Cao P, Yang Y (2023). Combination of RUNX1 inhibitor and gemcitabine mitigates chemo-resistance in pancreatic ductal adenocarcinoma by modulating BiP/PERK/eIF2α-axis-mediated endoplasmic reticulum stress. J Exp Clin Cancer Res.

[CR59] Sikdar S, Datta S (2017). A novel statistical approach for identification of the master regulator transcription factor. BMC Bioinformatics.

[CR60] Song J, Zhang X, Lv S, Liu M, Hua X, Yue L, Wang S, He W (2023). Age-related promoter-switch regulates Runx1 expression in adult rat hearts. BMC Cardiovasc Disord.

[CR61] Stone GW, Selker HP, Thiele H, Patel MR, Udelson JE, Ohman EM, Maehara A, Eitel I, Granger CB, Jenkins PL (2016). Relationship Between Infarct Size and Outcomes Following Primary PCI: Patient-Level Analysis From 10 Randomized Trials. J Am Coll Cardiol.

[CR62] Tang D, Kang R, Berghe TV, Vandenabeele P, Kroemer G (2019). The molecular machinery of regulated cell death. Cell Res.

[CR63] Tang PC-T, Chan MK-K, Chung JY-F, Chan AS-W, Zhang D, Li C, Leung K-T, Ng CS-H, Wu Y, To KF (2024). Hematopoietic Transcription Factor RUNX1 is Essential for Promoting Macrophage–Myofibroblast Transition in Non-Small-Cell Lung Carcinoma. Adv Sci.

[CR64] Tsao CW, Aday AW, Almarzooq ZI, Alonso A, Beaton AZ, Bittencourt MS, Boehme AK, Buxton AE, Carson AP, Commodore-Mensah Y (2022). Heart Disease and Stroke Statistics-2022 Update: A Report From the American Heart Association. Circulation.

[CR65] Turk B, Stoka V (2007). Protease signalling in cell death: caspases versus cysteine cathepsins. FEBS Lett.

[CR66] van Duijvenboden K, de Bakker DEM, Man JCK, Janssen R, Günthel M, Hill MC, Hooijkaas IB, van der Made I, van der Kraak PH, Vink A (2019). Conserved NPPB+ Border Zone Switches From MEF2- to AP-1-Driven Gene Program. Circulation.

[CR67] Xie Z, Zhao M, Yan C, Kong W, Lan F (2023). Narengaowa, Zhao S, Yang Q, Bai Z, Qing H et al: Cathepsin B in programmed cell death machinery: mechanisms of execution and regulatory pathways. Cell Death Dis.

[CR68] Xu G, Kanezaki R, Toki T, Watanabe S, Takahashi Y, Terui K, Kitabayashi I, Ito E (2006). Physical association of the patient-specific GATA1 mutants with RUNX1 in acute megakaryoblastic leukemia accompanying Down syndrome. Leukemia.

[CR69] Yadati T, Houben T, Bitorina A, Shiri-Sverdlov R (2020). The Ins and Outs of Cathepsins: Physiological Function and Role in Disease Management. Cells.

[CR70] Yang M, Liu E, Tang L, Lei Y, Sun X, Hu J, Dong H, Yang S-M, Gao M, Tang B (2018). Emerging roles and regulation of MiT/TFE transcriptional factors. Cell Commun Signal.

[CR71] Yuan J, Ofengeim D (2023). A guide to cell death pathways. Nat Rev Mol Cell Biol.

[CR72] Zhang J, Wang P, Huang YB, Li J, Zhu J, Luo X, Shi HM, Li Y (2010). Plasma cathepsin L and its related pro/antiangiogenic factors play useful roles in predicting rich coronary collaterals in patients with coronary heart disease. J Int Med Res.

[CR73] Zuzarte-Luis V, Montero JA, Torre-Perez N, Garcia-Porrero JA, Hurle JM (2007). Cathepsin D gene expression outlines the areas of physiological cell death during embryonic development. Dev Dyn.

